# 

**Published:** 1906-07-21

**Authors:** 


### BOOKS RECEIVED.

Everett and Co.

"The Elements of the Practice of Comparative Medicine." By F. T. Barton, M.R.C.V.S., and G. Gresswell, M.A.

Gale and Polden, Ltd.

"Guide to Promotion for Non-Coms. and Men of the R.A.M.C." By Captain S. T. Beggs.

E. and R. Garrould.

"The Midwife's Case Book." By Dr. C. St. Aubyn Farrer.

Macmillan and Co.

"The Prevention of Senility." By Sir James Crichton-Browne, M.D.

### NOTICE TO CORRESPONDENTS.

All MSS., Letters, Books for Review, and other matters intended for the Editor should be addressed The Editor, "The Hospital," The Hospital Buildings, 28 & 29 Southampton Street, Strand, W.C.

The Editor cannot undertake to return rejected MS., even when accompanied by stamped directed envelope.

### Notice to Advertisers and Subscribers.

All Advertisements, Orders for Copies of The Hospital, and Business Communications should be addressed to **THE MANAGER (Not to the Editor),** The Hospital, 28 & 29 Southampton Street, Strand London, W.C.

The Cost of Subscription, post free, is as follows:—Per week, 3½d.; per quarter, 3s. 3d.; per half-year, 6s. 6d.; per annum, 13s. Foreign and Colonial:—Per annum, 19s. 6d., payable, in all cases, in advance.

### POPULAR NURSING TEXT-BOOKS.

The works contained in this Series are well written by expert teachers of the subjects of which they treat, and will form a very valuable and useful addition to a Nurse's Library. Each volume is strongly bound in cloth cover, and contains about 100 pages.

Price 1/- each volume, post free.

**PRACTICAL HINTS ON DISTRICT NURSING.**
By Miss AMY HUGHES.

**HOSPITAL AND PRIVATE NURSING.**
By Miss S. E. ORME.

**THE MIDWIVES' POCKET BOOK.**
With Prefatory Chapter on the Midwives Bill.
By Miss HONNOR MORTEN, Author of "The Nurses' Dictionary," &c. &c.

**FEVERS AND INFECTIOUS DISEASES:**
Their Nursing and Practical Management.
By WILLIAM HARDING, M.D.Ed., M.R.C.P. Lond., Author of "Mental Nursing," &c.

**NOTES ON PHARMACY AND DISPENSING FOR NURSES.**
New and Revised Edition.
By C. J. S. THOMPSON.

**MENTAL NURSING.**
By WILLIAM HARDING, M.B. Ed., M.R.C.P. Lond.

**SICK NURSING AT HOME.**

By Miss L. G. MOBERLY.

**GYNÆCOLOGICAL NURSING.**
By G. A. HAWKINS-AMBLER, F.R.C.S. Author of "The Commonwealth of the Body."

**SYLLABUS OF LECTURES TO NURSES.**
By ANDREW DAVIDSON, M.D.

*JUST PUBLISHED.*

### THE CARE OF CHILDREN:

**Practical Hints for Mothers and Nurses at Home and Abroad.**

BY

**ROBT. J. BLACKHAM, D.Ph. (Lond.).**

1/6 net, 1/8 post free.

1/6 net. 1/8 post free.

Contains:—Hints on Food, Drink and Medicines, Clothing, Cleanliness, Rest and Exercise, Infectious Diseases, on the Nursery, on Nursery "First Aid," on Nursery Cooking, &c. &c.

**The Scientific Press, Ltd.,**

*Catalogue of Nursing Manuals sent post free on application.*

**Publishers of Nursing Text-Books, Charts, and Case-Books of all Descriptions.**

**28 & 29 SOUTHAMPTON STREET, STRAND, LONDON, W.C.**

### Publications.

### NEURASTHENIA, Clinical Lectures on.

By THOMAS D. SAVILL, M.D. Lond. 2nd Edition.

*Physician to the Hospital for Diseases of the Nervous System, &c. &c.*

"A timely and valuable contribution."—Journ. of Bal. & Climat.

"Deals very exhaustively with the subject."—The Lancet."

H. J. GLAISHER, 57 Wigmore Street, W. **5s. net; 5s. 4d. by post.**

**WORKS BY DAVID WALSH, M.D.**

**RÖNTGEN RAYS IN MEDICAL WORK.** *Third Edition.* 12s. 6d.

**THE HAIR AND ITS DISEASES.** 2s. 6d. net. A Standard Monograph. Baillère, Tindall, & Cox, London.

**GOLDEN RULES OF SKIN PRACTICE.** 1s. net. *Second Edition.* Wright & Co., Bristol.

**AGE AND OLD AGE: Handbook.** 2s. 6d. R. A. Everett & Co., London.

JUST PUBLISHED. Price **2s. 6d.** net.

**CARCINOMA OF THE RECTUM.**

By F. SWINFORD EDWARDS, F.R.C.S.,
Surgeon to the West London Hospital; Senior Surgeon to St. Peter's Hospital for Stone, and to St. Mark's Hospital for Fistula, &c.

London: Baillière, Tindall, & Cox, 8 Henrietta Street, Covent Garden.
THE MOST EFFECTUAL BANDAGE FOR THE CURE OF ULCERS AND OTHER DISEASES OF THE LEG.

**THE POCKET-BOOK OF TEMPERATURE CHARTS.**

Containing Seventeen 12-hour and eight 4-hour Charts, perforated so that each Chart may be easily removed after use.
Bound in paper cover. Suitable size for the pocket.
Price **6d.** net; by post **7d.**

*Of all Booksellers, or of*
THE SCIENTIFIC PRESS, LTD., 28 & 29 Southampton Street, Strand, London, W.C.

Now Ready. 10s. 6d. net; 10s. 11d. post free.

**The CONSUMPTIVE WORKING MAN:**

*WHAT CAN SANATORIA DO FOR HIM?*

BY

NOEL DEAN BARDSWELL,

M.D., M.R.C.P., F.R.S. (Edin.),

Medical Superintendent, King Edward VII. Sanatorium.

Of all Booksellers, or of

THE SCIENTIFIC PRESS, LTD.,

28/29 Southampton St., Strand, London, W.C.

